# A Case Report on Genital Tuberculosis

**DOI:** 10.7759/cureus.30548

**Published:** 2022-10-21

**Authors:** Riddhi Lele, Deepti Shrivastava

**Affiliations:** 1 Obstetrics and Gynaecology, Jawaharlal Nehru Medical College, Datta Meghe Institute of Medical Sciences, Wardha, IND

**Keywords:** cheesy material, fallopian tubes, infertility, tubal blockage, genital tuberculosis

## Abstract

Female genital tuberculosis is a significant cause of female infertility. It has been noted that about 10% of females having infertility were due to genital tuberculosis (TB). It is an accidental finding while one is investigating infertility. Laparoscopy is the investigation of choice. A histopathologic examination is done to confirm the diagnosis. A 34-year-old primigravida with 35 weeks + four days gestation with IVF conception and decreased fetal movements with ultrasonography suggestive of a double loop of cord around the neck came for safe confinement. She was operated on July 6, 2022, as an emergency lower segment cesarean section procedure with bilateral fimbriectomy. The indication was that this was an IVF baby, and the mother noticed diminished fetal movements. She was discharged on July 11, 2022. A color doppler was done on the day of discharge which showed normal findings. The patient was asked to come for a follow-up after 15 days in the outpatient department or so in case of an emergency. A high protein and iron diet, plenty of fluids, and adequate rest were recommended. Symptoms differ depending on the severity and spread of the disease. Typical symptoms of TB include fever, night sweats, and weight loss. Other presentations include menstrual dysfunction, and chronic abdominal pain, with or without general symptoms like weight loss. Genital TB can cause abdominal adhesions and tubal blockage. It even causes infertility and menstrual abnormalities like amenorrhea and menorrhagia in females. The investigations to be done are the Mantoux test, chest X-ray, and cartridge-based nucleic acid amplification test (CBNAAT) to detect the presence of *tubercle bacilli*. Hysterosalpingography is done to check for infertility. A laparoscopy is to be performed. Symptomatic management of the patient can lead to a successful pregnancy.

## Introduction

As far as India's condition is concerned, the prevalence of tuberculosis is very high. It is still an endemic disease in India, with its major focus on the poorer sections. Socioeconomic factors like overcrowding and low income can precipitate the disease. Tuberculosis affecting any organ in the body has a primary origin, that is, lungs; it then spreads to the fallopian tubes, which is considered a hot spot of genital tuberculosis. The disease can spread hematogenously or via lymphatics to the affected part. When it affects the fallopian tubes, it leads to the oozing of cheesy material in response to inflammation. Genital tuberculosis can present with weight loss, and menstrual abnormalities like amenorrhea are common.

Fallopian tubes are affected most commonly, one can appreciate various pathologies related to them like peri-salpingitis, tubo-ovarian masses, and tubercular peritonitis. Tuberculous salpingitis leads to a granuloma on the surface of the tubes [1]. Hysterosalpingography (HSG), and laparoscopy, are some of the investigations to be performed. Oviduct obstruction is the most common HSG finding in genital tuberculosis [2]. The definitive diagnosis of genital tuberculosis depends on a positive culture of mycobacteria [3]. Patients with HIV infection are most vulnerable to tuberculosis [4]. The polymerase chain reaction can be falsely positive. It is not enough alone to make a diagnosis [5].

Investigations to diagnose tuberculosis need to be carried out. They include the Mantoux test and hysterosalpingography (HSG). Typical HSG findings are beads-on-string appearance, lead pipe appearance and tobacco-pouch appearance. Once the diagnosis is made, the disease should be managed aggressively with drugs and surgery.

Various treatment modalities are available. It includes general treatment, chemotherapy, and surgical option. Antitubercular treatment is chemotherapy, consists of four drugs in the initial phase, which are Isoniazid (300mg), Rifampicin (600mg), Pyrazinamide (1.5 grams/day), Ethambutol (1200 mg/day) for four months and three drugs in the continuation phase which are Isoniazid, Rifampicin, and Ethambutol for four months [6]. The general treatment implies hospital admission in the case of pulmonary tuberculosis. Bed rest is recommended for the patient with pelvic TB. The surgical option is to be tried only when the patient is unresponsive to chemotherapy or develops chronic pain or persistent menorrhagia. The surgery done is a fimbriectomy. 

## Case presentation

**Figure 1:**
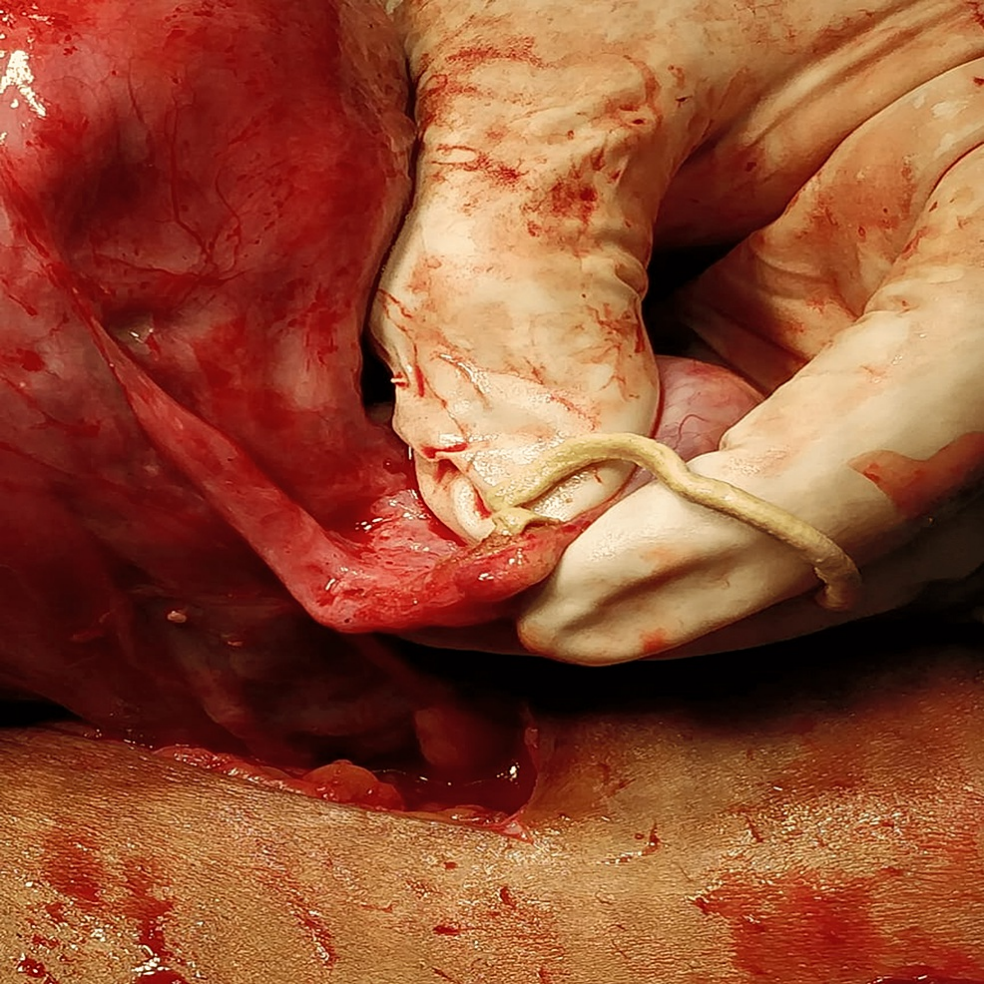
Cheesy material from fallopian tube

**Figure 2:**
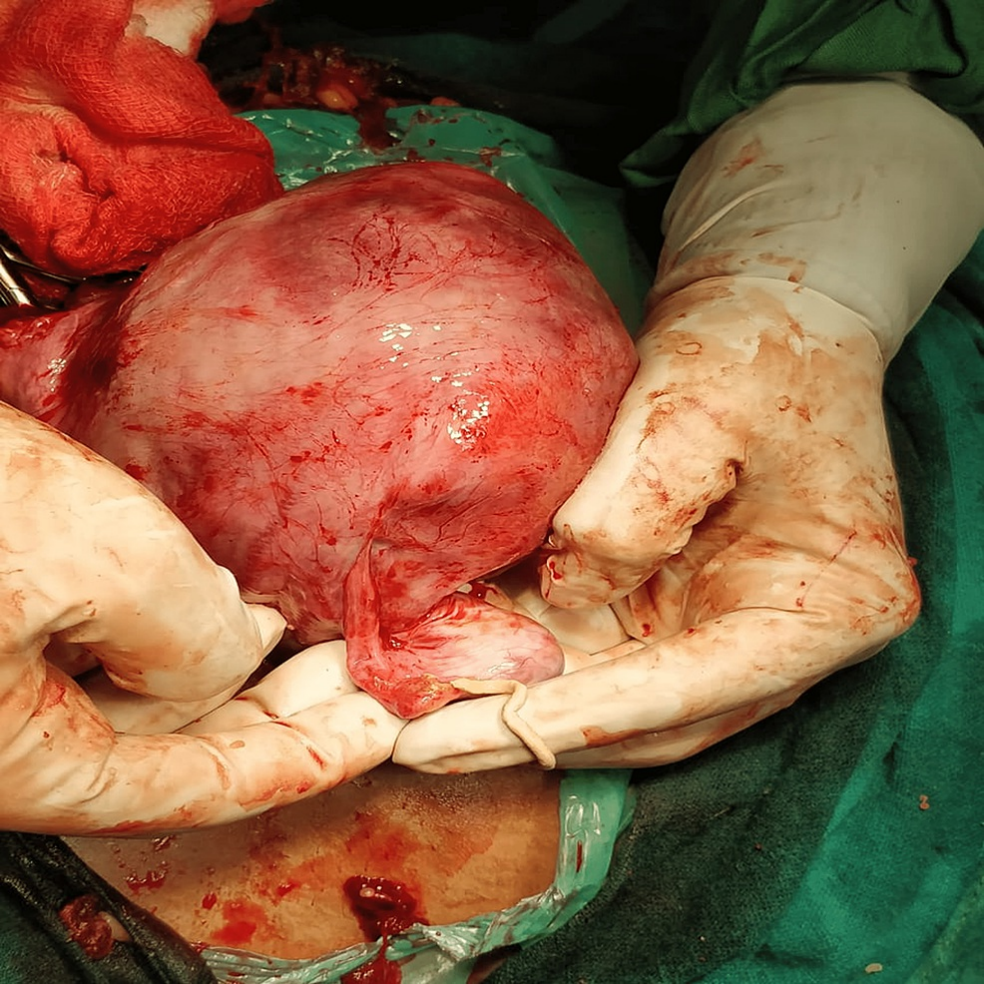
On pressing the fallopian tube, cheese like material oozes out.

We present a case of a 34-year-old female patient, a resident of Wardha district, Maharashtra, Hindu by religion, who came to the rural hospital outpatient department (OPD) on July 2, 2022, with eight months amenorrhea and chief complaint of decreased fetal movements for one day. Her gestational age was 35 weeks + four days with IVF conception and decreased fetal movements with ultrasonography suggestive of a double loop of cord around the neck for safe confinement. She has been married for six years and is a primigravida. The mother took two tetanus toxoid injections. Her menstrual history shows her last menstrual period was on October 26, 2021, and the IVF specialist estimated her expected delivery date to be August 2, 2022. Her premenstrual history is a period lasting for around 4-5 days every 30 days, regular with the normal flow, with no history of clots or dysmenorrhea. She has a history of taking in vitro fertilization (IVF) treatment since 2018. The history of diagnostic hysterolaparoscopy done in 2018 was suggestive of blocked tubes. There is a history of hysterosalpingography done for the same. Hysteroscopic findings on October 4, 2019, showed the presence of both left and right Ostia. When the disease affects the fallopian tubes, it leads to the oozing of cheesy material (Figures 1, 2).

Laparoscopic results showed the cavity appearing normal, blind end tube with no fimbriae, left tube adherent to the peritoneum of the sigmoid colon, a right ovary is atrophic with a tubo-ovarian mass, fluid reduced in the pouch of Douglas. Investigations also included a specimen from fallopian tubes, which was sent for histopathological examination and showed caseous cheesy material coming from both tubes. The hormone test showed serum Anti-Mullerian Hormone (AMH) levels of 3.42ng/ml, Follicle-Stimulating Hormone (FSH) levels of 6.34, and luteinizing hormone (LH) levels of 2.89 done on 8^th^ March 2021. The IVF specialist noted serum AMH levels to be 0.69ng/ml on June 24, 2021. The husband's semen analysis which the lab specialist did on May 5, 2021, showed the amount of 2ml, a count of 60-70 million, motility of 70% (grade 4), and morphology of 80% (good). There is no specific illness associated. History is not significant, with no history of diabetes mellitus or hypertension, Bronchial asthma, tuberculosis, thyroid disorder, or epilepsy. No previous history of hospital admission. There is no history of blood transfusion. The doctor took the patient consent. On general examination, the patient was conscious, cooperative, and well-oriented to time, place, and person. She has an average build. Height is 160 cm. Her weight is 60 kg, so her body mass index (BMI) turns out to be 23.4. She is afebrile to touch with a pulse rate of 88beats/minute, respiratory rate of 16 cycles per minute, and blood pressure of 110/70mm of hg. All the systemic examinations were under normal limits. The abdominal examination showed a uterus corresponding to a 34-week size. Fetal heart sounds were present, which were 140 beats per minute. On investigations, the blood group is B positive. The HIV test is negative. 

On doing complete blood count (CBC), hemoglobin is 10.8%, normocytic normochromic, platelets reduced in number, and no hemoparasites were seen. Normal fetal growth and monitoring and serial ultrasonography (USG) were done, one of which was done on July 2, 2022, which suggested a live intrauterine fetus of 35 weeks and six days corresponding to a weight of 2795 grams with amniotic fluid index (AFI) of 15 with a double loop of cord around the neck. Normal color doppler flow and spectral waveform. She was prescribed supplements like iron, calcium, and vitamins for routine management. The Foley catheter was inserted pre-operatively and was removed on the second post-operative day, after which the mother passed urine.

## Discussion

Tuberculosis is a disease primarily seen in the poorer sections of society. Genital tuberculosis occurs after the infection from the lungs spreads to the fallopian tubes via the distant spread. One can appreciate pulmonary as well as genital tract symptoms in such patients. The woman experiences menstrual problems like amenorrhea, which later predisposes her to infertility. The bacilli, after affecting the lungs, affect the fallopian tubes, where they cause infection. The bacilli can cause inflammation at the site of infiltration. If the disease is aggressively spreading, it can cause perforation of the tubes. A major cause of the destruction of fallopian tubes is caseous necrosis caused by the acid-fast bacilli. Various modalities can diagnose genital TB. Chest roentgenogram, Tuberculin test, and hysterosalpingography should be performed. Endometrial tissue should be extracted by curettage and sent for histopathological study [7]. Negative culture from urine curetting does not exclude the diagnosis of genital TB.

Hysterosalpingography is the most crucial investigation to diagnose infertility [8]. Aggressive drug therapy should be carried out in such patients, followed by fimbriectomy, which means the removal of fallopian tubes if they are involved to a greater extent. Even after this, there are chances that the infection destroys the fallopian tubes, thereby making it not favorable for a normal pregnancy. Therefore, in such patients, IVF is recommended. Treatment with IVF proves to give results in infertile women. 

## Conclusions

From the above discussion on genital tuberculosis, it is clear that a woman will have to face the problem of infertility because the probability of conceiving by a natural method is almost equal to zero. Not only the woman suffers from menstrual abnormalities, but there is the development of adhesions in the endometrium, which make the uterus an unfavorable environment. Diagnosing genital tuberculosis at an early stage with medical treatment, which includes drugs, can prevent the development of infertility. So, in vitro fertilization turns out to be her best option. This process somewhat takes a longer time, but the outcome is promising.
